# An approach for determining and measuring network hierarchy applied to comparing the phosphorylome and the regulome

**DOI:** 10.1186/s13059-015-0624-2

**Published:** 2015-03-31

**Authors:** Chao Cheng, Erik Andrews, Koon-Kiu Yan, Matthew Ung, Daifeng Wang, Mark Gerstein

**Affiliations:** Department of Genetics, Geisel School of Medicine at Dartmouth, Hanover, New Hampshire USA; Institute for Quantitative Biomedical Sciences, Geisel School of Medicine at Dartmouth, Lebanon, New Hampshire USA; Norris Cotton Cancer Center, Geisel School of Medicine at Dartmouth, Lebanon, New Hampshire USA; Program in Computational Biology and Bioinformatics, Yale University, 260 Whitney Avenue, New Haven, CT 06520 USA; Department of Molecular Biophysics and Biochemistry, Yale University, 260 Whitney Avenue, New Haven, CT 06520 USA; Department of Computer Science, Yale University, 260 Whitney Avenue, New Haven, CT 06520 USA

## Abstract

Many biological networks naturally form a hierarchy with a preponderance of downward information flow. In this study, we define a score to quantify the degree of hierarchy in a network and develop a simulated-annealing algorithm to maximize the hierarchical score globally over a network. We apply our algorithm to determine the hierarchical structure of the phosphorylome in detail and investigate the correlation between its hierarchy and kinase properties. We also compare it to the regulatory network, finding that the phosphorylome is more hierarchical than the regulome.

**Electronic supplementary material:**

The online version of this article (doi:10.1186/s13059-015-0624-2) contains supplementary material, which is available to authorized users.

## Background

Networks have been used as universal frameworks to represent many complex systems including the World Wide Web [1], social interactions [2], literature citation relationships [3], and biological processes [4-6]. Based on the attributes of edges, networks can be subdivided into two categories: undirected and directed. In an undirected network there is no distinction between the two vertices associated with each edge, whereas in a directed network all edges are directed from one vertex to another. The asymmetric nature of edges in a directed network causes topological differences of nodes, resulting in a hierarchical structure: some function as top regulators, while others function as downstream effectors.

Owning to the development of large-scale experimental techniques, many biological networks have been produced. These include protein-protein interaction networks and genetic interaction networks [7-12]. Among them, the gene regulatory network (referred to as the regulome) and the protein phosphorylation network (referred to as the phosphorylome) are two of the best-studied directed networks [10,11]. The regulome captures the transcriptional regulatory interactions of transcription factors (TFs) with their target genes. The techniques to systematically identify TF-DNA interactions include the bacterial one-hybrid system [13], the yeast one-hybrid system [14], and chromatin immunoprecipitation followed by microarray (ChIP-chip) [15] or parallel sequencing (ChIP-seq) [16]. In particular, ChIP-chip and ChIP-seq have been used to determine the target genes of a large number of TFs in recent years, and will produce more data in the near future. In particular, in yeast Harbison *et al.* have performed ChIP-chip experiments to identify target genes of 203 proteins, which represent nearly all of the DNA-binding transcriptional regulators encoded in the yeast genome [10]. In human, the Encyclopedia of DNA Elements (ENCODE) project has determined the genomic binding sites of more than 120 TFs [17]. Meanwhile, the interactions between kinases, phosphotases, and their substrates can be identified by protein chip [11] or mass spectrometry [18]. The latter technology is capable of providing precise phosphorylation sites. In particular, Ptacek *et al*. has determined the *in vitro* substrates recognized by most yeast protein kinases [11]. The availability of these datasets enables us to construct regulomes and phosphorylomes and investigate the regulatory mechanisms of TFs and kinases on a systems level.

Since the regulome and phosphorylome are directed networks, it is of particular interest to examine whether they harbor a hierarchical structure (TF/kinase nodes function at different levels) and, if so, how that hierarchy is organized. Particularly, we have previously investigated the rewiring of the regulomes in *E. coli* and *S. cerevisiae*, and found that hierarchy, rather than connectivity, better reflects the importance of regulators [19]. For the regulomes, the hierarchy properties have been explored in several studies [17,20-23]. In these studies, the authors inferred the hierarchical structure of regulomes and examined the correlation between the hierarchy and TF features. For example, Jothi *et al*. demonstrated that top-level TFs in the yeast regulome are more likely to be essential and are more conserved across species [21]. These studies provide critical insights into the regulatory mechanisms of TFs during transcription regulation. On the other hand, the phosphorylation network has not been investigated from a hierarchical perspective.

Several algorithms have been proposed to infer the hierarchical structure of directed networks [20,21,24-27], including leaf-removal, breadth-first-search (BFS) and vertex sort (VS) methods. These algorithms have been applied to the regulome and revealed new insights on hierarchical organization of TFs during transcriptional regulation. Despite their effectiveness, they have several limitations and do not address some important issues about hierarchical networks. The leaf-removal algorithm determines the hierarchical structure by removing leaves iteratively, and as a consequence it cannot be applied to a directed network with cycles. Similarly, for networks with cycles the BFS method has to break cycles before assigning hierarchical levels to nodes [20]. In addition, these two methods do not allow any ambiguity in positioning a node among hierarchical level - a feature that is often the case in many networks. The VS algorithm proposed by Jothi *et al*. is capable of overcoming these shortcomings. However, it can only assign ambiguous nodes to an interval of potential levels without providing the probability of them to be at each level [21].

Moreover, these methods have not addressed several important questions related to hierarchical networks: How to quantify the degree of hierarchical structure for a given network? How to estimate significance of hierarchical structure of a directed network? How to compare the degree of hierarchy of two directed networks? Importantly, can every node be assigned to a specific level with the same confidence? If not, how can we know which nodes are more confident than the others? For those ambiguous nodes, what are the probabilities of them to be assigned to each level?

The degree of hierarchy for a given for a given network is not well-defined concept. Ispolatov *et al.* [27] introduced the idea of dominant direction by minimizing the number of feedback links. While it is a proxy of hierarchical structure to a certain extent, the method does not provide a rigorous statistical confidence. Here, we define a metric to quantify the degree of hierarchy for a given hierarchical network, and then propose a new method called hierarchical score maximization (HSM) to infer the hierarchy of a directed network. First, we apply the algorithm to a military command network which possesses a perfect hierarchical structure. The results demonstrate its effectiveness in precisely determining the network’s hierarchy. Second, we apply the algorithm to eight directed networks including biological networks, social networks, and ecological networks. We compare these networks in terms of their degrees of hierarchy and the results suggest that phosphorylomes are more hierarchical than transcriptional regulatory networks. Third, we compare the hierarchical structure of the yeast regulome determined using the HSM algorithm with those from previous algorithms. Finally, we investigate the hierarchical structure of the yeast phosphorylome in detail and relate kinases in different levels with different genomic features.

## Results

### Construction of hierarchy by simulated annealing

**Figure 1:**
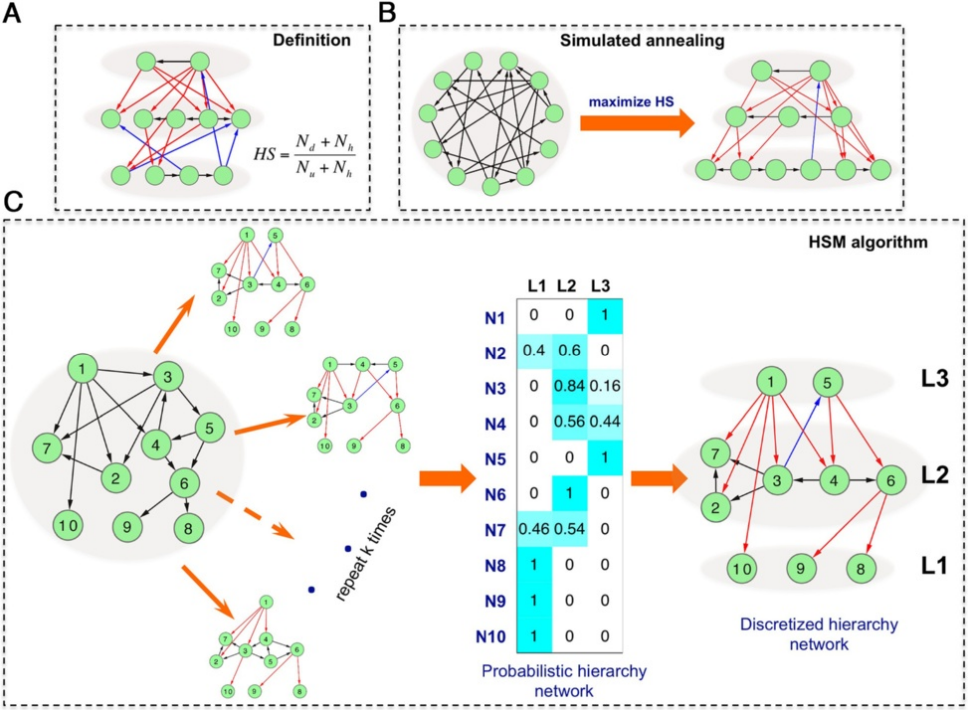
The schematic diagram of the hierarchy score maximization algorithm. In hierarchical networks, the downward, upward, and horizontal edges are shown in red, blue, and black colors, respectively. (**A**) The definition of hierarchy score. (**B**) A simulated annealing algorithm for inferring the hierarchical structure by maximizing the hierarchy score. (**C**) The procedure to calculate the probability of nodes in different hierarchy levels. Simulated annealing procedure is performed for k runs and in each run a hierarchical structure is inferred by maximizing the hierarchy score. The frequency of each node in these k hierarchical networks is calculated to obtain a probabilistic hierarchical network. Discretized hierarchical network is obtained by assigning nodes to the level with highest frequency.

To infer the hierarchical structure of a directed network, we start by defining a score to quantify the degree of hierarchy. For a network with a specified hierarchical topology (that is, every node is assigned to a specific hierarchy level), there are in general three types of edges: downward interactions (pointing from higher-level to lower-level nodes), upward interactions (pointing from lower-level to higher -level nodes), and horizontal interactions (between nodes in the same level). We thus define the hierarchy score (HS) as the ratio of the number of downward interactions (N_d_) to the number upward interactions (N_u_) balanced by the number of horizontal interactions (N_h_) (see ‘Materials and methods’ for details) (Figure 1A). Based on this definition, we infer the hierarchical structure of a directed network as the one that achieves the maximum hierarchy score. Specifically, a simulated annealing algorithm is used to continuously adjust the hierarchical structure until the hierarchy score is maximized (Figure 1B). Since HS will increase as the number of levels is increased, the HS for two hierarchical networks with different numbers of levels are in general not directly comparable. To address this issue, we therefore elaborate HS into a new metric called the corrected hierarchy score (CHS), which quantifies the enrichment in downward flow relative to expectation (see ‘Materials and methods’ for details). Finally, we define a *P* value for how likely one would get such a hierarchical structure randomly.

In principle, the optimum hierarchical structure for a directed network may not be unique due to the existence of loops. Some nodes can be assigned to different levels without significant change of hierarchy score. For this reason, it is more reasonable and more informative to represent the hierarchical structure as a probabilistic model, in which a node may be assigned to multiple levels with different probabilities. To estimate these probabilities, for a directed network we performed the simulated annealing procedure 1,000 times (k = 1,000); aggregating the results from each run gives rise to probabilistic assignments to the different levels (Figure 1C). Accordingly, we define a score called the probabilistic hierarchy score (PHS) to more accurately quantify the hierarchy underlying a directed network (see ‘Materials and methods’ for details). Typically, most of the nodes have a favored level to which the node is assigned with a significantly higher probability than the other levels. We thus can obtain a determined hierarchical structure by assigning each node to its most likely level (Figure 1C).

### Application of the HSM algorithm to a military command network

**Figure 2:**
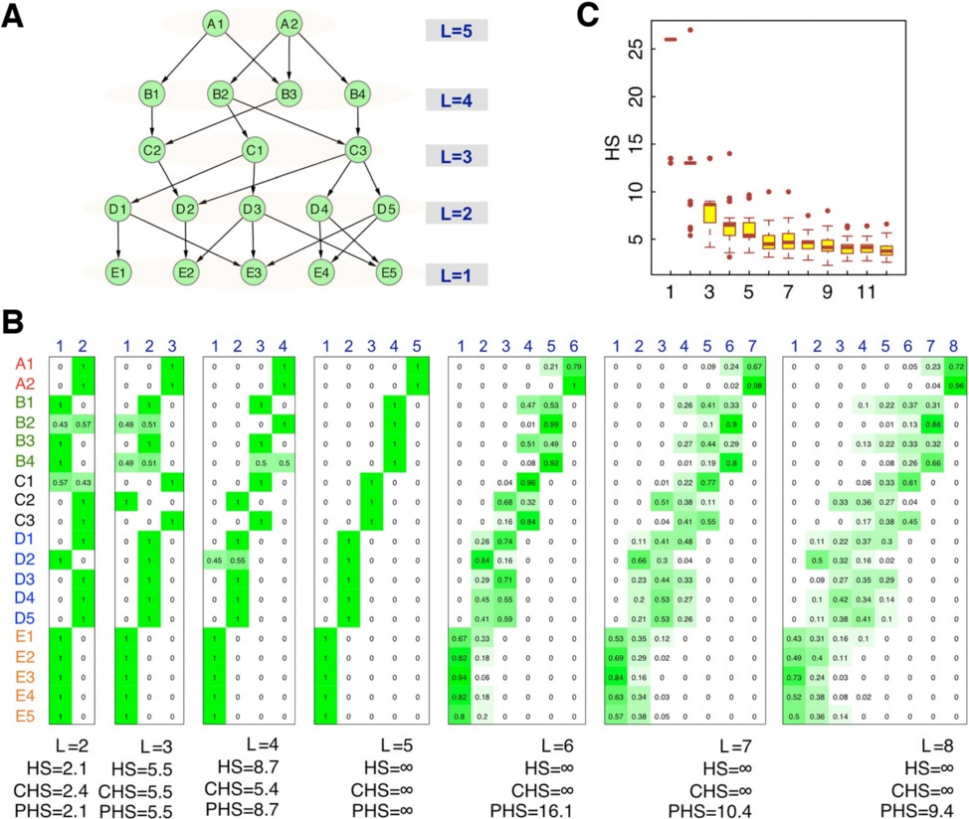
Application of the hierarchy score maximization algorithm to a military command network. (**A**) A military command network with 19 nodes at five hierarchy levels. (**B**) The probability matrix inferred by the HSM algorithm with the number of levels specified as L = 2, 3, …, 8. Each element in the matrix gives the probability of a node being assigned to a level. The HSM algorithm correctly identifies the network hierarchy when L = 5 is specified. (**C**) The distribution of hierarchy scores when a certain number of edges in the original network are perturbed. HS: hierarchy score; CHS: corrected hierarchy score; PHS: probabilistic hierarchy score (see ‘Materials and methods’ for details).

To show the effectiveness of our method we apply it to a military command network, which we know is a directed acyclic graph (DAG) with a perfect hierarchical structure. Since there are no loops in the network, the hierarchy levels of each node can be deterministically assigned (Figure 2A). We then apply the HSM algorithm to the network, specifying different number of levels L = 2, 3, …, 8. As shown in Figure 2B, the hierarchical structure is precisely inferred when the correct number of levels (L = 5) is specified. All of the nodes are assigned to the right levels with 100% certainty. Meanwhile the largest HS, CHS, and PHS were obtained when L is set to 5. In practice, we do not have the prior knowledge about the number of hierarchical levels. To determine the number of levels one can try a range of different L values and then set L to a ‘saturating value’ so that further increase of L results in no or little increase in CHS and PHS.

To show that the HS can quantify the degree of hierarchy of a directed network we perturb the original network in Figure 1A by introducing a number of upward edges. We randomly introduce a number of upward edges (n) to perturb the hierarchical structure of the network. For each n, we repeated our procedure and re-determined the hierarchical structure of the perturbed networks. As shown in Figure 2B, with the increase numbers of perturbations the hierarchy scores of the perturbed networks decrease asymptotically, indicating that the HS is an effective measurement for quantifying the degree of hierarchy of directed networks.

### Hierarchical scores of several directed networks

We next apply the HSM algorithm to calculate the degree of hierarchy of eight different directed networks, including five biological networks (yeast regulatory network, human regulatory network, yeast phosphorylation network, human phosphorylation network and worm neural network), one ecological network (food web), one social network (political blogs), and one computer network (P2P file sharing network) (see ‘Materials and methods’ for information about these networks). We can evaluate the performance of the HSM algorithm, since we have an intuitive sense of the degree of hierarchy of these non-molecular networks.

In Table 1, we summarize the topological properties of these eight networks being sorted in the increasing order of CHSs. The political blog network contains hyperlinks between weblogs on US politics being recorded in 2005 [3]. The weblogs refer to each other by hyperlinks largely in a non-hierarchical manner, and consistently, we observe a relatively low hierarchical structure of it (CHS = 3.2). In contrast, the food web network typically is known to have a pyramidal structure: the number of predators at each level decreases significantly, so that a single top predator is supported by a much larger number of preys. Indeed, the food web network is more hierarchical, with a CHS of 6.4. In addition, the worm neural network is the least hierarchical one among these networks, consistent to our knowledge that neurons are not hierarchically but mutually connected with one another [28].

**Table 1:** **Hierarchical scores of eight directed networks**

**Network**	**Nodes (n)**	**Edges (n)**	**Levels (n)**	**1-DR**	**KHS**	**GRC**	**HS**	**CHS**	**PHS**	**Significance** ^**a**^	**Reference**
Worm neural	297	2,359	L = 4	0.184	0.186	0.133	2.805	2.364	2.707	1.60 (30.72)	Watts *et al.* [45]
Political blogs	1,224	19,087	L = 3	0.487	0.514	0.130	3.044	3.177	2.972	2.23 (251.38)	Adamic *et al.* [3]
Yeast TF	149	580	L = 4	0.559	0.611	0.381	4.675	3.869	4.330	1.97 (22.32)	Harbison *et al.* [10]
Human TF	112	513	L = 4	0.631	0.718	0.336	7.087	5.608	5.848	3.15 (53.30)	Gerstein *et al.* [23]
P2P file sharing	6,301	20,777	L = 4	0.486	0.772	0.628	4.348	5.878	2.401	1.71 (74.06)	Ripeanu *et al.* [58]
Foodweb	63	612	L = 3	0.259	0.261	0.582	5.788	6.407	5.788	4.26 (190.38)	Ulanowicz *et al.* [46]
Human kinase	373	2,171	L = 4	0.492	0.798	0.020	14.087	13.396	12.874	7.16 (275.89)	Newman *et al.* [38]
Yeast kinase	94	200	L = 4	0.645	0.775	0.447	17.455	13.982	11.777	4.81 (41.73)	Ptacek *et al.* [11]

^a^Significance is calculated by comparing a network with 1,000 Erdos-Renyi random networks. The first number is the ratio of its HS to the average HS of the random networks. The second number in the parenthesis is the t-statistics of the HS. All networks listed in the table are significant (*P* <2e-16).

1-DR: 1-dyadic reciprocity; CHS: corrected hierarchy score; GRC: global reaching centrality; HS: hierarchy score; KHS: Krackhardt hierarchy score; PHS: probabilistic hierarchy score.

**Figure 3:**
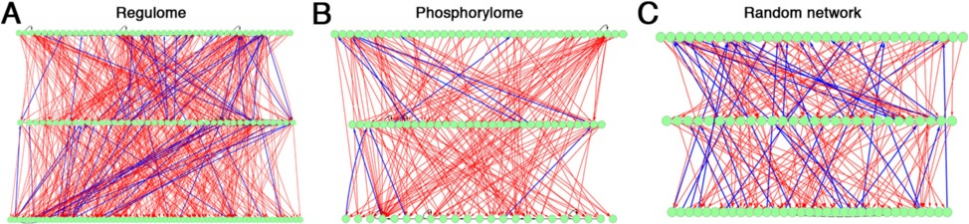
Application of the HSM algorithm to the yeast regulome (**A**), phosphorylome (**B**), and a random network (**C**).

Our results reveal several interesting findings. First, in both human and yeast, the phosphorylome is more hierarchical than the regulome (Table 1 and Figure 3), although all of these networks show significant hierarchical structures compared to a random network (*P* <2e-16, see method for significance estimation). This is seen with the corrected hierarchy scores (CHSs) for yeast regulome and human regulome of 3.9 and 5.6, respectively, in contrast to the CHSs for yeast phosphorylome and human phosphorylome of 13.4 and 14.0, respectively. Surprisingly, the phosphorylomes are even more hierarchical than the food web network. Strikingly, all previous hierarchical network studies have been focused on regulomes and overlooked the phosphorylomes [17,20,21]. Our findings suggest that more investigation into the hierarchical nature of phosphorylome is warranted. Second, the degrees of hierarchy for both regulome and phosphorylome are highly consistent between yeast and human, two evolutionarily distant species.

### Comparison with other hierarchy construction algorithms

To compare the HSM algorithm with other methods, we apply it to the yeast regulome which contains 580 regulatory interactions among 145 transcription factors. With the same dataset, Yu *et al.* have applied a BFS method to construct a four-level hierarchical network [20]; Jothi *et al.* have applied a vertex sort (VS) approach to obtain a hierarchical network with seven levels, and further merged them into three levels [21]. We execute the HSM algorithm and obtain hierarchical networks with 3, 4, …, 8 levels. According to the CHSs, the hierarchical network with four levels is the most appropriate one.

**Figure 4:**
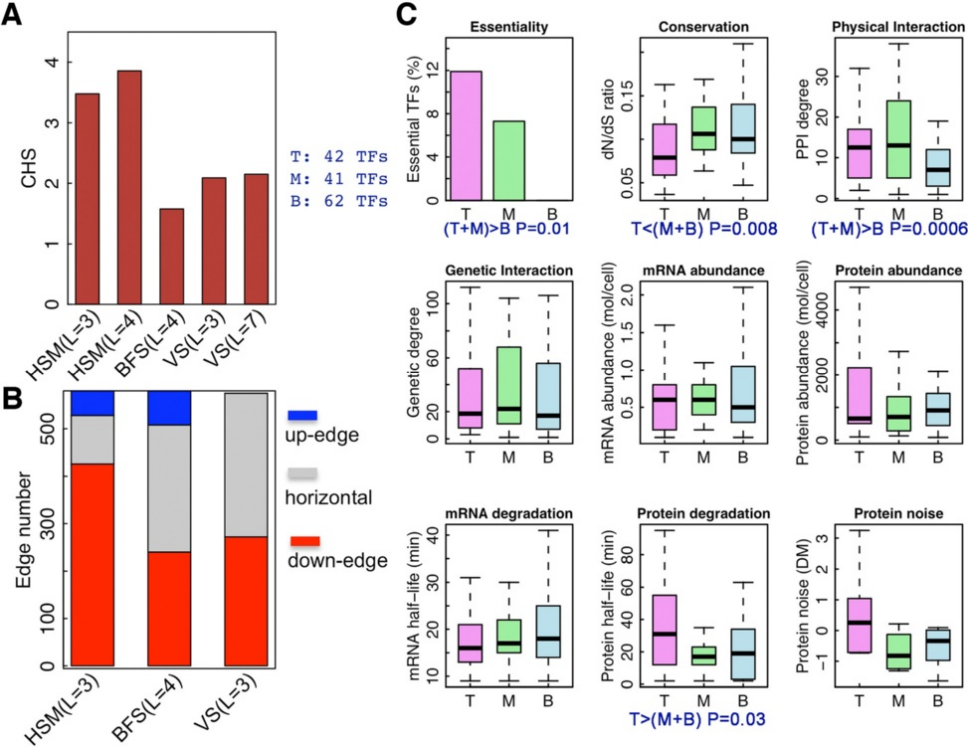
Application of HSM algorithm to the yeast regulome. (**A**) The corrected hierarchy scores for hierarchical networks as inferred by HSM, BFS and VS methods. (**B**) The number of downward, upward, and horizontal edges in hierarchical networks inferred by the three methods. (**C**) The correlation of TF properties with hierarchy. T, M, and B represent top, middle, and bottom levels, respectively.

We compare the CHSs of the hierarchical networked inferred by different methods (Figure 4A). As HSM is designed to maximize the hierarchical score it gives rise to networks with significantly higher CHSs than those by BFS and VS methods (Figure 4A). The hierarchical networks inferred by the other two methods have much lower CHSs than the optimum score. Moreover, the hierarchical network inferred by the HSM algorithm shows the highest fraction of downward interactions with >70% of interactions pointing from higher to lower level TFs. This is in contrast to BFS and VS where <50% of interactions are downward. Although there are no upward interactions in the hierarchical network derived from the VS algorithm (L = 3), it has more horizontal interactions than the HSM algorithm (Figure 4B). A similar fraction of horizontal edges are observed in the seven-level hierarchical network inferred by the VS algorithm.

We next examine the properties of TFs in relation to the hierarchy inferred by the HSM algorithm. As shown in Figure 4A, the hierarchical network for the yeast regulome with four levels (L = 4) achieves the highest CHS, but the CHS for the network with three levels (L = 3) is just slightly lower. In order to simplify the downstream analysis and to facilitate the comparison with previous studies we focus our analysis on the one with three TF levels, with 42, 41, and 62 TFs at the top, middle, and bottom levels, respectively (Additional file 1: Table S1). First, we compare the percentage of essential TFs in the three levels. Our results indicate that higher level TFs are more likely to be essential: five out of 42 top level TFs (12%) and three out of 41 middle level TFs (8%) are essential. In contrast, none of the 62 bottom level TFs is essential (*P* = 0.01, Fisher’s exact test). In line with this, the TFs at the higher levels are more conserved during the evolution with the top level TFs tend to having a lower dN/dS ratio (calculated based on *S. cerevisae* versus *S. pombe* comparison) than the middle and bottom level TFs (*P* = 0.008, Wilcoxon rank sum test). These results are consistent with those previously reported in Jothi *et al.* [21]. Second, we examine the degrees of the TFs at different levels in the physical interaction and genetic interaction networks. We find that TFs in higher levels (T + M) have significantly more physical interactions (*P* = 0.0006, Wilcoxon rank sum test) than those in the bottom level, consistent with our observations in the human regulome [17]. The average numbers of partners for TFs in different layers rank in the order of M > T > B. A similar trend (M > T > B) is observed for genetic interactions, but it does not pass the significance threshold (*P* >0.05 when TFs in T + M layers are compared to those in B layer in terms of number of genetic interactions). Third, we compare the TFs at different levels on their dynamic properties, including their abundance and stability at both the mRNA and protein level, and their protein expression noise. The results indicate that the top-level TFs are more stable than middle- and bottom-level TFs (*P* = 0.03, Wilcoxon rank sum test) (Figure 4C). Overall, our results highlight the critical roles played by the top-level TFs, as also reported by Jothi *et al.* using the VS algorithm [21]. These master regulators are highly conserved during evolution with a higher essentiality rate.

### Features of kinases at different levels

Our results suggest that the organization of phosphorylomes is more hierarchical than the regulomes. We infer the hierarchical structure of the yeast phosphorylome by using the HSM algorithm. This network is mainly based on protein chip experiments and contains 200 phosphorylation interactions among 94 different kinases [11,29]. Again, for easy comparison we specify the number of hierarchical levels L = 3, which results in 38 top-level, 33 middle-level, and 23 bottom-level kinases (Additional file 2: Table S2).

**Figure 5:**
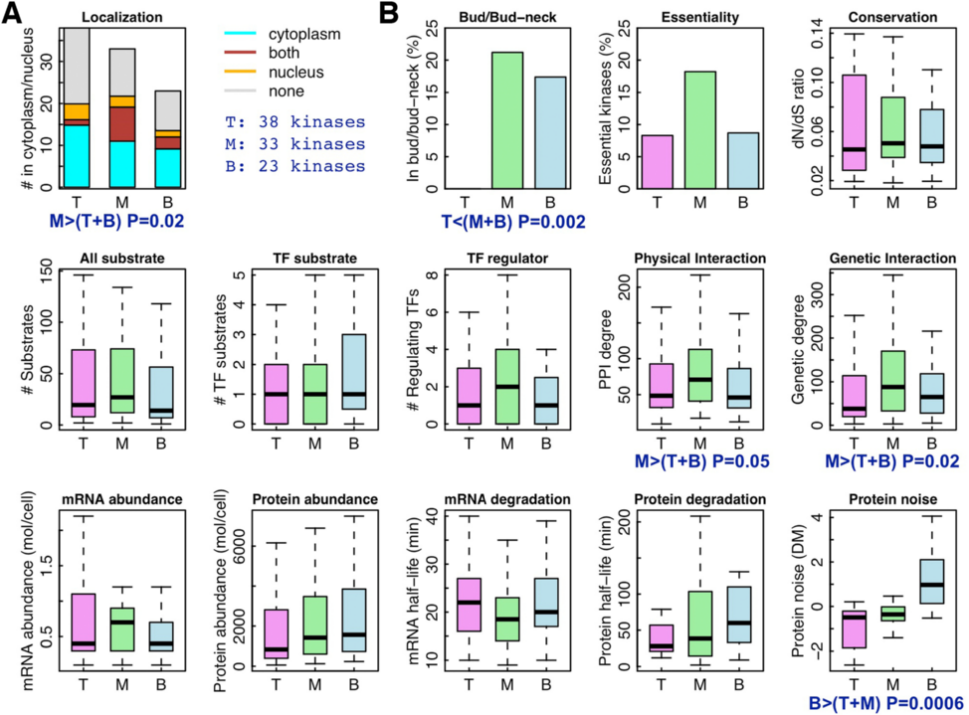
Application of the HSM algorithm to the yeast phosphorylome. (**A**) The localization of kinases at different levels in the cytoplasm and nucleus. (**B**) The correlation of kinase properties with hierarchy. T, M, and B represent top, middle, and bottom levels, respectively.

We examine the cellular localization according to the Saccharomyces genome database, which are manually annotated based on previous literatures. Of the 94 kinases 35 localize only to the cytoplasm, eight only to the nucleus, and 12 to both (the remaining 39 kinases are in other locations or localization unknown). Interestingly, the kinases in the middle level are more likely to localize in both nucleus and cytoplasm compared to the top and bottom level kinases (*P* = 0.02, Fisher’s exact test, Figure 5A). Gene ontology analysis suggests that the top level is enriched in trans-membrane proteins and stress-response proteins implying that the top-level kinases tend to be located in the cell membrane and respond to extracellular signals (Additional file 3: Table S3). In contrast, the middle level is enriched in cell cycle related kinases.

We also relate the hierarchical structure of the yeast phosphorylome with a number of kinase properties (Figure 5B). Our findings are summarized as follows: (1) The bud/bud-neck located proteins are highly enriched in kinases of the middle and bottom levels with respect to the top level (*P* = 0.002, Fisher’s exact test). Strikingly, none of the 38 top-level kinases is a bud/bud-neck protein. This may suggest that during yeast budding the top-level kinases function mainly in the mother cells rather than enter the bud/bud-neck to perform as direct effectors. (2) The middle-level kinases show higher essentiality rate (18%) than the top-level (8%) and the bottom-level (8%) kinases. (3) Kinases in the middle level have significantly more physical (*P* = 0.05, Wilcoxon rank sum test) and genetic (*P* = 0.02, Wilcoxon rank sum test) interaction partners. (4) The bottom-level kinases are significantly noisier in their protein abundance than kinases in the higher levels (*P* = 0.006, Wilcoxon rank sum test).

### Collaboration of kinases in different levels

**Figure 6:**
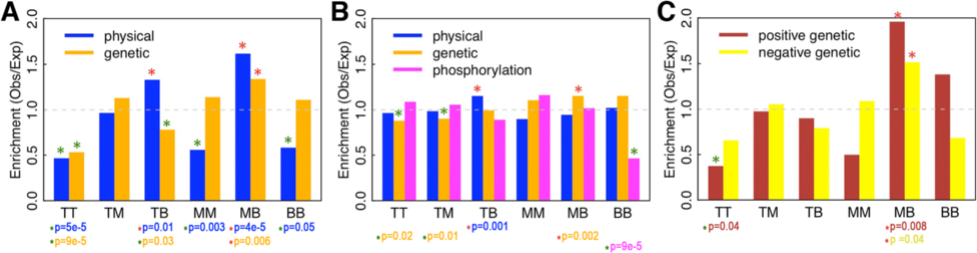
Collaboration of kinases at different hierarchy levels. (**A**) The enrichment of physical and genetic interactions of kinases within a level (TT, MM, and BB) and between two levels (TM, TB, and MB). (**B**) The enrichment of kinase pairs with significantly overlapping physical or genetic interaction partners or phosphorylation substrates. (**C**) The enrichment of positive and negative genetic interactions of kinases. Enrichment and depletion of interactions (*P* <0.05) are marked as red and green ‘*’, respectively.

We next explore how kinases in the top, middle, and bottom levels collaborate with one another, in terms of both inter-level (TM, MB, TB) and intra-level (TT, MM, BB) relationships. First, we examine the physical and genetic interactions between kinases at different levels. Our results show that physical interactions are significantly enriched in TB (between top-level and bottom-level kinases) and MB (between middle-level and bottom-level kinases), but depleted in the intra-level relationships (TT, MM, and BB). The genetic interactions are significantly enriched in MB, and depleted in TT and TB relationships (Figure 6A). This suggests that inter-level interactions between kinases, particularly between middle- and bottom-level kinases, are dominant over those intra-level interactions.

Second, we investigate kinase cooperativity. We define two kinases as being cooperative if they share a significantly large number of physical partners, genetic partners, or phosphorylation substrates (Figure 6B). We find that physical cooperation between kinases is enriched in TB, while genetic cooperation is enriched in MB relationships. Interestingly, cooperation is highly depleted between bottom level kinases suggesting that, as downstream effectors, they tend to phosphorylate different subsets of proteins to take specific effects. Finally, we further divide genetic interactions into positive and negative ones, and examine their enrichment or depletion between kinases. Positive and negative genetic interactions involve a pair of genes with mutations or deletions in which each alone causes a minimal phenotype, but when combined in the same cell results in a less severe (positive) or a more severe (negative) fitness defect than expected under a given condition [30]. As shown in Figure 6C, both positive and negative genetic interactions are significantly enriched in MB relationships.

### Substrate of kinases at different levels

**Figure 7:**
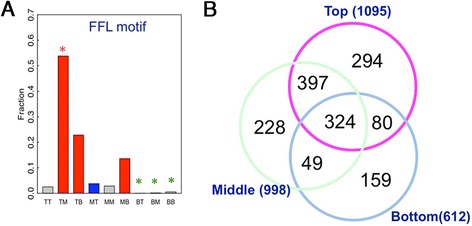
Properties of the inferred hierarchical structure for the yeast phosphorylome generated by HSM algorithm. (**A**) The distribution of feed-forward loop (FFL) motifs in the hierarchical network. In a FFL motif, a kinase X phosphorylates another kinase Y and both target a common substrate Z. Depending on the location of X and Y in the hierarchical structure, the X-- > Y interaction can be categorized into nine combinations. Downward interactions (TM, TB, and MB), upward interactions (MT, BT, and BM), and horizontal interactions (TT, MM, and BB) are shown with red, blue, and gray bars, respectively. (**B**) The Venn diagram of substrates of kinases at different levels.

The network contains 200 inter-kinase phosphorylation interactions (one kinase phosphorylating another) and six auto-phosphorylation interactions (CKA2, TPK2, RAD53, PRP1, CDC7, and CDC15). Indeed, the auto-phosphorylation is over-represented in the network (*P* = 0.02, see ‘Materials and methods’ for details). There are two feedback loops (TPK2 and TPK3, ELM2 and GIN4) involving two kinases in the network in which the two kinases phosphorylate each other. The feed-forward loop (FFL) network motif is highly enriched in the yeast phosphorylome. We investigate the FFL motifs in the context of hierarchy. In a FFL with three nodes, one kinase phosphorylates another kinase and both target a third protein as substrate, which can be either a kinase or non-kinase. We enumerate all of three-node FFL motifs in the yeast phosphorylation data (including non-kinase substrates) and map the two kinases in these motifs to the hierarchical network. Each of the two kinases in a FFL motif can be from one of the three hierarchical levels (T, M, and B), which results in nine combinations (TT, TM, TB, MT, MM, MB, BT, BM, and BB). We count the number of FFL motifs for all the nine types and our results show that >90% FFL motifs involve downward interactions between kinases in the hierarchical networks (Figure 7A, red bars). The TM type FFL motif, in which a top-level kinase phosphorylates a middle-level kinase and both kinases share a target substrate, is significantly enriched.

We also examine and compare the functions of the substrate targets of kinases at different levels. The 38 top-level kinases target a total of 1,095 substrates; the 33 middle-level kinases target 998 substrates; and the 23 bottom-level kinases target 612 substrates. The substrate targets of the three levels highly overlap as shown in Figure 7B. After filtering out the shared substrate targets, we identify 294 top-level, 228 middle-level, and 159 bottom-level specific substrate targets. Gene ontology analysis indicates that the top-level specific substrates are enriched in gene categories involving in ‘protein kinase activity’, ‘phosphorylation’, and ‘phosphate metabolic process’, and so on (Additional file 4: Table S4). In other words, the top-level kinases are involved in the regulation of other phosphorylation-related proteins. In contrast, the middle- and bottom-level specific substrate targets are enriched in structural proteins, for example, gene categories involving in ‘microtubule cytoskeleton’, ‘structural molecule activity’, and ‘macromolecular complex subunit organization’.

## Discussion

### Global optimization versus local optimization

To determine the hierarchical structure of a directed network, the leaf removal and the BFS methods apply a local optimization strategy. The leaf removal algorithm employs a bottom-up iterative procedure. It assigns all the leaf nodes (nodes with zero out-degree) to the bottom level, and then iteratively removes all the leaf nodes and the edges associated with them from the network to determine the next higher level [25]. The BFS method also starts by assigning the leaf nodes to the bottom level, and then performs a BFS to define the level of a non-bottom node as its shortest distance from a bottom one [20]. In contrast, the hierarchical score maximization (HSM) algorithm presented here works to globally optimize the hierarchy of a directed network. It defines a hierarchy score (HS) to quantify the degree of hierarchy in a network. The hierarchical score captures the global hierarchical property of a network. To infer the hierarchy, HSM optimizes the hierarchical structure so that the maximum HS is achieved. Thus, the hierarchy inferred by HSM represents the globally optimized structure. The VS algorithm identifies strongly connected components and collapses them to convert the network into a directed acyclic graph, and applies the leaf removal algorithm on the graph and on its transpose. Results are then combined to infer a global solution of hierarchical levels [21]. This method avoids any upward edges but the resulting networks have smaller hierarchy scores compared to those from the HSM algorithm.

Compared with the previous methods, the HSM algorithm takes into account the potential hierarchical ambiguity underlying a network. It provides a probabilistic representation of the hierarchy for a network that can more precisely reflect the underlying hierarchical structure. For all the nodes, we know the certainty of them being assigned to a hierarchical level, which is informative and is useful for us to interpret their roles in the hierarchical network. A global optimization method has been proposed in [27], which applied a simulated annealing algorithm to minimize the number of ‘backward’ links going from lower to higher hierarchical levels. In contrast, we define a hierarchical score that quantifies the degree of hierarchy and infer the hierarchical structure of a network by the scores magnitude.

Moreover, the HSM algorithm’s corrected hierarchical score (CHS) is comparable between different networks. As shown in Table 1, this enables comparisons in the degree of hierarchy between different biological networks such as social networks, file sharing networks, ecological networks, and neural networks. Practically, this allows for the exploration of the common rules shared by different networks and reveals the differences between them [31]. For example, we find that the protein phosphorylation interactions mediated by kinases are much more hierarchical than the transcriptional regulatory interactions mediated by TFs.

### Hierarchy versus asymmetry for directed networks

Dyadic reciprocity and Krackhardt hierarchy score are often used to quantify the extent of asymmetry in directed networks [32]. The former is defined as the proportion of node pairs that are reachable from either direction, while the latter is the fraction of node pairs that are reachable from only one direction. We note that Krackhardt hierarchy score, though termed a ‘hierarchy’ score, is distinct from the hierarchy score (HS) described here. The asymmetry measured by reciprocity or Krackhardt score quantifies the degree to which two nodes are ‘mutually reachable’ in a directed network. A related metric called global reaching centrality (GRC) was defined to measure hierarchy as heterogeneous distribution of the local reaching centrality (the proportion of all nodes that can be reached from a node) of all nodes in a directed network [33]. These metrics do not imply any information on orientation. In contrast, by hierarchy here we mean a top-to-bottom orientation for nodes at different levels.

Why do we need to introduce the ‘orientation/hierarchy’ attribute for a directed network? Because in many networks the nodes are by nature associated with certain ‘spatial’ or ‘temporal’ attributes. For example, the protein nodes in a biology network may localize in different cellular components, for example, the membrane, cytoplasm, or nucleus; meanwhile, external signals are often transduced following a specific direction from membrane to nucleus. This confers a global ‘hierarchy’ attribute to the network that cannot be captured by ‘asymmetry’ attributes (for example, reciprocity and Krackhardt score). On the other hand, since the ‘hierarchy’ originates from certain attributes of nodes, we would expect to observe the correlation of hierarchy with node features. In other words, the inferred hierarchical structure should recapitulate the attribute difference of nodes at different levels. For instance, as shown in Figure 4, we find that the higher-level TFs in the yeast regulome are more likely to be essential and more conserved.

The hierarchy score is also different than the three-dimensional ‘morphospace’ proposed recently by Corominas-Murtra, which defines three hierarchical features: treeness, feed-forwardness, and orderability [34]. To define them, nodes with zero in-degrees and out-degrees are regarded as the source and the sink of a network, respectively, and then the paths between them are characterized.

### Temporal versus spatial organization of hierarchy

We observe interesting results when we apply the HSM algorithm to the yeast regulome and phosphorylome. In the yeast regulome, we find that higher-level TFs are more likely to be essential and are more conserved during evolution. Particularly, none of the 62 bottom-level TFs are essential, compared to an average essentiality rate of 19% in yeast. In the yeast phosphorylome, however, this is not the case and instead we observe significant differences in cellular localization for kinases at different levels. For instance, 21% of the middle-level and 18% of the bottom-level kinases are detectable in bud/bud-neck, whereas in the 38 top-level kinases, none are identified in bud/bud-neck.

Biologically, the hierarchy of regulatory networks (regulome and phosphorylome) may arise from the temporal and/or spatial organization of regulators. In response to stimulation or in a biological process (for example, cell cycle regulation), early-activated regulators (for example, TFs or kinases) regulate the expression/activation of later regulators, which in turn regulate even later ones, forming a hierarchical structure. Similarly, the cellular localization of regulators can also contribute to the hierarchical organization of a regulatory network. For example, during signal transduction the extracellular signal is typically transferred from a membrane-localized kinase to a cytoplasmic kinase and onward to a nuclear kinase [35]. Since in general TFs function in the nucleus by regulating gene expression, their hierarchy is mainly organized via temporal activation of TFs. However, in phosphorylomes the hierarchical organization of kinases can be determined by both temporal regulation and spatial localization. The differential correlation pattern of protein features with hierarchy between regulomes and phosphorylomes may reflect such a difference.

One caveat of this study involves the quality of data used for constructing the phosphorylomes. The yeast phosphorylome is constructed based on two sources: kinase-substrate interactions identified from protein chip experiments by Ptacek *et al.* [11] and phosphorylation site data collected by Freschi *et al.* from several large-scale studies [36]. The Patcek data are derived from *in vitro* measurements of kinase-substrate interactions, some of which may not necessarily occur *in vivo*. The Freschi data derived from a set of experimentally identified phosphorylation sites, for which the associated protein kinases were computationally predicted by matching with position weight matrices of yeast kinases. Thus, we would expect a high false positive rate in the yeast phosphorylome, and, similarly, this is also the case for human phosphorylome. More confident kinase-substrate interactions might be obtained by selecting those in which kinase and substrate are present in the same cellular compartment or in the same functional categories. However, this filtering procedure may also increase the number of false negatives since the protein localization data and function annotation may be incomplete or inaccurate. In addition, only a subset of kinase-substrate interactions have been identified and included in the phosphorylomes. Therefore, a more detailed analysis should be performed to validate findings in this analysis when more complete and accurate phosphorylation data become available in the future.

## Conclusions

In summary, the HSM algorithm provides a useful tool to investigate the hierarchy of directed networks. It can be used independently or in conjunction with other hierarchy inference methods. With more and various regulatory interaction data being generated, we expect a wide application of these methods in biological network studies.

## Materials and methods

### Construction of network hierarchy

A hierarchical network is a directed network for which all nodes are assigned to a unique hierarchical level from 1 to L, where L is the total number of levels (L ≥2). Generally, a hierarchical network contains three types of edges according to their directionality: a downward edge (pointing from a higher level node to a lower level node), an upward edge (pointing from a higher level node to a lower level node), and a horizontal edge (pointing from a node to another node in the same level). To infer the hierarchy of a directed network, we developed a hierarchical score maximization algorithm described as follows.

First, given a directed network with assigned hierarchical structure, we define a metric called hierarchy score as:

$HS=\frac{N_d+{N}_h}{N_u+{N}_h}$, where N_d_, N_u,_ and N_h_ are the number of downward edges, upward edges, and horizontal edges, respectively. The metric essentially measures the ratio of N_d_ to N_u_ balanced by N_h_. It takes a value from 0 to + ∞, with a higher HS indicating more downward edges relative to upward edges in a network. Specifically, when N_u_ = N_h_ = 0, the network will have a HS of + ∞.

Second, for a directed network we employ a simulated annealing procedure [37] to infer its hierarchical structure by arranging nodes into L levels (L is a pre-defined parameter). This procedure is as follows:

1. We initiate from randomly assigning each node to a level, calculate the corresponding HS score hs_0_, and setting the initial energy as E_0_ = −hs_0_.
2. We adjust the hierarchy iteratively to optimize the hierarchical structure. Specifically, at iteration i, we randomly select a node, adjust the hierarchy by randomly placing it into another level and recalculate the hierarchical score and energy (hs_i_ = −E_i_) of the resulting new hierarchy. We compute the energy change ∆E = E_i_-E_i-1_; if ∆E < 0, we accept the hierarchy adjustment; otherwise we accept the adjustment with a probability *P* = exp(−∆E/CT), where C is a constant and T is temperature that are used to tune the probability *P*.
3. We repeat this procedure p times until E is minimized (that is, HS is maximized). In practice, we gradually lower the temperature T at each step to adjust the sensitivity of annealing. This procedure results in an optimized hierarchical network with maximized HS score.

Third, we perform the above-described simulated annealing algorithm k = 1,000 times to obtain 1,000 inferred hierarchical networks. We do this because in many cases the optimum hierarchy is not unique. For example, some nodes are topologically identical in a directed network, and changing their level assignment coordinately will not change the overall hierarchical score. Based on these 1,000 inferred hierarchical networks, we calculate the probability that each node is assigned to each level, which results in a probability matrix for each node as seen in Figure 1C. This matrix can be regarded as a probabilistic hierarchical network, which is more informative and more precisely describes the hierarchical structure of a directed network than methods that omit this procedure.

Fourth, we provide a most likely hierarchical network based on the probabilistic hierarchical matrix. Specifically, we assign each node to the level for which the prior step assigns it the highest probability. It should be noted that the confidence of the assignment might vary from node to node, depending on the value of the maximum probability. Typically, however, most of the nodes have high certainty in terms of the level assignment (for example, the probability in the assigned level is >60%).

To determine an appropriate p (the number of steps in each simulated annealing procedure) and k (the number of each simulated annealing runs), we plot the hierarchy score against p and k, respectively. For a network with more nodes and edges, a larger p should be used as can be determined based on the HS vs. p plot. When a suitable p is used, the resulting HS should be stable against k when k is >100.

In practice, the HSM method can be used conjunction with other hierarchy inference methods. For example, one may start from the hierarchical structure inferred by the VS algorithm, and use the simulated annealing procedure method to further optimize the hierarchy score. Namely, instead of randomly selecting nodes during the simulated annealing optimization, we can focus on adjusting the levels of ambiguous nodes from VS output to improve the efficiency. Such a strategy will combine the advantages of the two hierarchy inference approaches.

### Determination of the number of hierarchical levels

The HSM algorithm requires a pre-defined L, the number of hierarchical levels. L can be determined based on the prior knowledge about the directed network of interest. If no prior knowledge is available, we can specify different L values (e.g. L = 2, 3, …, 8) and choose a proper L by comparing the resulting hierarchical networks. However, the HS score is not directly comparable for hierarchical networks with different number of levels, because networks with larger L tend to have higher HSs. We thereby define a corrected hierarchical score (CHS) as the following:

$$ CHS=\frac{O\left({N}_d\right)/E\left({N}_d\right)+O\left({N}_h\right)/E\left({N}_h\right)}{O\left({N}_u\right)/E\left({N}_u\right)+O\left({N}_h\right)/E\left({N}_h\right)}, $$

where O(N_d_), O(N_u_), and O(N_h_) are the observed number of downward, upward, and horizontal edges, respectively; E(N_d_), E(N_u_), and E(N_h_) are the expected number of downward, upward, and horizontal edges, respectively. E(N_d_), E(N_u_), and E(N_h_) are calculated as:

$$ E\left({N}_d\right)={\displaystyle \sum_{i>j}{S}_i{S}_j}; $$

$$ E\left({N}_u\right)={\displaystyle \sum_{i<j}{S}_i{S}_j}; $$

$$ E\left({N}_h\right)={\displaystyle \sum_{i=j}{S}_i{S}_j}, $$

where S_i_ and S_j_ are the number of nodes in level *i* and level *j*, respectively. The CHS is directly comparable between hierarchical networks with different L values, and can also be used to compare the degree of hierarchy between different directed networks. The CHS takes a value from 1 for random network without a hierarchical structure to ∞ for a network with a perfect hierarchy (for example, a tree as in Figure 2).

To determine the number of hierarchical level L for a network, one can employ the HSM algorithm across a range of L values and choose the L for which the HSM algorithm yields the highest CHS. In some cases, the CHS will keep increasing with the increase of L, because there is more freedom to optimize the hierarchy with larger L values. In this situation, one can plot the CHS against L values, and choose the L at which no significant CHS improvement is achieved. In addition, other information is also important to determine the L for a directed network. For example, it is reasonable to require L to be no larger than the diameter of the network, namely, the greatest distance between any pair of connected nodes.

### Calculation of probabilistic hierarchical score

To more accurately measure the hierarchical structure of a probabilistic hierarchical network, we define a new metric called the probabilistic hierarchical score (PHS). For an edge *i → j* in a network with L levels, the probability of this edge being downward is ${\displaystyle {\sum}_{L_i>{L}_j}P\left({L}_i,i\right)P\left({L}_j,j\right)}$, where *P*(*L*_*i*_, *i*) and *P*(*L*_*j*_, *j*) are the probability of the node i and j in level L_i_ and L_j_, respectively. Similarly, the probability of *i → j* to be upward is ${\displaystyle {\sum}_{L_i<{L}_j}P\left({L}_i,i\right)P\left({L}_j,j\right)}$; and the probability of *i → j* to be horizontal is ${\displaystyle {\sum}_{L_i<{L}_j}P\left({L}_i,i\right)P\left({L}_j,j\right)}$. Thus after taking into account all edges in the network, we define PHS as the following:

$$ PHS=\frac{{\displaystyle {\sum}_{\left(i\to j\right)\in \left\{e\right\}}{\displaystyle {\sum}_{L_i>{L}_j}P\left({L}_i,i\right)P\left({L}_j,j\right)}}+{\displaystyle {\sum}_{\left(i\to j\right)\in \left\{e\right\}}{\displaystyle {\sum}_{L_i={L}_j}P\left({L}_i,i\right)P\left({L}_j,j\right)}}}{{\displaystyle {\sum}_{\left(i\to j\right)\in \left\{e\right\}}{\displaystyle {\sum}_{L_i<{L}_j}P\left({L}_i,i\right)P\left({L}_j,j\right)+{\displaystyle {\sum}_{\left(i\to j\right)\in \left\{e\right\}}{\displaystyle {\sum}_{L_i={L}_j}P\left({L}_i,i\right)P\left({L}_j,j\right)}}}}}. $$

The level is indexed in an increasing order from bottom to top. Namely, level *i* is higher than level *j* in the hierarchy, if *i > j*.

### Estimation of the hierarchy significance for a directed network

Given a directed network, the HSM algorithm infers its optimum hierarchical structure by maximizing the HS score. Although the resulting HS score can measure the degree of hierarchy of a network, it does not tell us whether a directed network has a significantly hierarchical structure. To address this issue, we compare a directed network with random networks to evaluate its hierarchical significance. Here we use the Erdos-Renyi random graph model as the null model. In a network, each pair of nodes has an equal chance to be connected by an edge [38]. We generate 1,000 Erdos-Renyi random networks with the same number of nodes and edges, and calculate their HSs using the HSM algorithm. Given a specified number of layers (L), the *P* value of hierarchy for the network of interest is then computed as the fraction of random networks with a HS (the same L is specified) equal to or greater than the interested network. Alternatively, assuming a Gaussian distribution of the HSs of the random networks, we calculate the Z-score for the interested network: Z = (HS-μ)/σ, where μ and σ are the mean and standard deviation of the HS scores of those random networks with L levels; the *P* value is calculated by referring to a standard normal distribution, and for each L a corresponding *P* value is calculated (L = 2, 3, …, 8). The hierarchy for the whole network is calculated as the minimum P-value adjusted by the Bonferroni multiple-testing correction method. We note that the significance estimation depends on the selection of the null model. To generate the random networks, other null models can be used and certain constraints can be applied as required.

### Calculation of dyadic reciprocity and Krackhardt hierarchy score

Traditionally, 1-dyadic reciprocity and Krackhardt hierarchy score are often used to quantify the extent of asymmetry in directed network [32]. The dyadic reciprocity is defined as the proportion of node pairs in a directed network that are symmetric (that is, reachable from either direction). Krackhardt hierarchy score is the fraction of node pairs in the directed network that are reachable from one direction. These two metrics measure the degree of asymmetry of a directed network, which is different from the hierarchy we introduce in this study. Our hierarchy by nature implies a top-to-bottom orientation, whereas the ‘asymmetry’ is non-directional. We use the R package ‘sna’ to calculate dyadic reciprocity and Krackhardt hierarchy score. The global reaching centrality of networks is calculated using the method introduced by Mones *et al.* [33].

### Directed networks used in this study

In this study, we examine eight directed networks, including five biological networks, one ecological network (food web network), one social network (political blogs network), and one computer network (P2P file sharing network). The five biological networks are the yeast regulation network, the human regulation network, the yeast phosphorylation network, the human phosphorylation network, and the worm neural network.

The yeast regulome was downloaded from Jothi *et al.* [21], in which most of the TF-gene interactions were identified by ChIP-chip experiments [9,10], and the remaining were collected based on other biochemical studies [39-42]. The human regulome is constructed based on ChIP-seq data from the ENCODE project [43], based on which the target genes of more than 120 TFs are determined by a probabilistic model [44]. For TFs with multiple ChIP-seq datasets, the target genes represent a union of targets in all of the available datasets. The kinase-substrate interactions in the yeast phosphorylome are collected from protein chip experiment by Ptacek *et al.* [11] and the phosphorylation site data collected by Freschi *et al.* from several large-scale studies [36]. The human phosphorylome is available from the PhosphoNetworks database, which is based on experimental determined kinase-substrate relationships [38]. In our hierarchical study, we include only TF-TF interactions in the two regulomes and kinase-kinase interactions in the two phosphorylomes.

The worm neural network contains the interaction of one neuron to another via synaptic or gap junctions in worm [45]. The food web network is from Ulanowicz *et al.*, which contains the carbon exchange from one species to another occurring during the wet season in the cypress wetlands of south Florida [46]. The Political blogs network contains hyperlinks between weblogs on US politics being recorded in 2005 [3]. The P2P file-sharing network is one of a series of Gnutella network created in 2002, in which nodes represent host computers in the Gnutella computer network and edges represent connections between the hosts [47].

### Properties of yeast genes and proteins

The list of yeast essential genes was determined by a yeast gene deletion project and was downloaded from the Saccharomyces genome database (SGD) [48]. The Ka/Ks ratios of ortholog genes between S. *cerevisiae* and S. *pombe* orthologs were from Wall et al. [49]. The physical and genetic interactions of yeast genes were also downloaded from the SGD database [50,51]. Specifically, to calculate the number of physical interacting partners of yeast kinases the protein-protein interactions between yeast kinases were obtained from Breitkreutz *et al*. [12]. The mRNA abundance and mRNA half-life data were obtained from previous studies [52,53]. The protein half-life data came from Belle *et al*. [54]. The protein abundance and protein noise data were available from Newman *et al*. [55]. To determine the protein noise, the single cell expression level of a protein was measured in a population of yeast cells and then the ratio of the standard deviation to its mean abundance was calculated. For a protein, the noise is represented as the difference between its noise value and the median over all proteins, named as deviation from median (DM). Budding or budding neck localization of yeast kinases was obtained from Huh *et al.* [56]. The cellular component associated with yeast kinases was annotated by SGD, which are manually curated based on previous publications.

### Enrichment of interactions between different levels

To examine whether TFs/kinases are more likely to physically/genetically interact within the same level or between two levels (Figure 6A), we calculated the enrichment of interactions in all pairs of levels: TT, TM, TB, MM, MB, and BB. Using physical interactions between TFs as the example, the significance of enrichment or depletion is calculated as follows. First, given a physical interaction network with n nodes and e edges, we compute the probability for a pair of randomly selected genes to interact: p = e/[n(n-1)/2]. Second, we assume that the number of TF-TF interactions (denoted as *i*) within a level or between two different levels follows a binomial distribution: $\Pr \left(x=i\right)=f\left(i;b,p\right)={C}_b^i{p}^i{\left(1-p\right)}^{b-i}$, where b is the number of all possible TF-TF pairs. Considering self-interactions, b = m(m + 1)/2 for intra-level interactions with m TFs (that is, TT, MM, or BB), and b = m_1_m_2_ for interactions between two levels with m_1_ and m_2_ TFs, respectively (that is, TM, TB, and MB). Finally, the *P* values are calculated as P(x ≥ *i*) for enrichment (that is, the probability of observing an equal or greater number of interactions) and P(x ≤ *i*) for depletion (that is, the probability of observing an equal or smaller number of interactions) of physical interactions between these TFs.

To estimate whether two kinases share a significantly large number of physical partners, genetic partners or substrates (Figure 6B), we examine their degree of overlap and calculate its significance using Fisher’s exact test (that is, hyper-geometric test).

### Gene ontology analysis

We used the DAVID Gene Ontology (GO) annotation tool [57] to investigate the functional enrichment of kinases in the three levels of our hierarchical network for phosphorylome (Figure 3B). The whole list of the 94 kinases in the network is used as the background for enrichment analysis. A similar analysis is also used to study the functional enrichment of substrates specific to kinases from each of the three levels. In this case, we use the whole yeast gene list as the background. R code for this analysis is available from http://gersteinlab.org/proj/hinet.

## Additional files

Additional file 1: Table S1. The hierarchical structure of the yeast regulome with three levels: 3 - Top, 2 - Middle, 1 - Bottom. Additional file 2: Table S2. The hierarchical structure of the yeast phosphorylome with three levels: 3 - Top, 2 - Middle, 1 - Bottom. Additional file 3: Table S3. GO analysis results of kinases in the top, middle, and bottom levels. Additional file 4: Table S4. GO analysis results of specific substrate proteins of kinases in the top, middle, and bottom levels.
